# Self-rated health in Pakistan: results of a national health survey

**DOI:** 10.1186/1471-2458-5-51

**Published:** 2005-05-19

**Authors:** Khabir Ahmad, Tazeen H Jafar, Nish Chaturvedi

**Affiliations:** 1 Clinical Epidemiology Unit, Department of Community Health Sciences, Aga Khan University, Karachi, Pakistan; 2 Section of Ophthalmology, Department of Surgery, Aga Khan University, Karachi, Pakistan; 3 Section of Nephrology, Department of Medicine, Aga Khan University, Karachi, Pakistan, and Division of Nephrology, Department of Medicine, Tufts-New England Medical Center, Tufts University Medical School, Boston, MA, USA; 4 Department of Epidemiology and Public Health, Imperial College of Medicine at St. Mary's, UK

## Abstract

**Background:**

Self-rated health (SRH) is a robust predictor of mortality. In UK, migrants of South Asian descent, compared to native Caucasian populations, have substantially poorer SRH. Despite its validation among migrant South Asian populations and its popularity in developed countries as a useful public health tool, the SRH scale has not been used at a population level in countries in South Asia. We determined the prevalence of and risk factors for poor/fair SRH among individuals aged ≥15 years in Pakistan (n = 9442).

**Methods:**

The National Health Survey of Pakistan was a cross-sectional population-based survey, conducted between 1990 and 1994, of 18 135 individuals aged 6 months and above; 9442 of them were aged ≥15 years. Our main outcome was SRH which was assessed using the question: "Would you say your health in general is excellent, very good, good, fair, or poor?" SRH was dichotomized into poor/fair, and good (excellent, very good, or good).

**Results:**

Overall 65.1% respondents – 51.3 % men vs. 77.2 % women – rated their health as poor/fair. We found a significant interaction between sex and age (p < 0.0001). The interaction was due to the gender differences only in the ages 15–19 years, whereas poor/fair SRH at all older ages was more prevalent among women and increased at the same rate as it did among men. We also found province of dwelling, low or middle SES, literacy, rural dwelling and current tobacco use to be independently associated with poor/fair SRH.

**Conclusion:**

This is the first study reporting on poor/fair SRH at a population-level in a South Asian country. The prevalence of poor/fair health in Pakistan, especially amongst women, is one of the worst ever reported, warranting immediate attention. Further research is needed to explain why women in Pakistan have, at all ages, poorer SRH than men.

## Background

South Asia, home to over 1.5 billion people (a quarter of the world's population) has one of the worst health indicators in the world [1]. Around 34% of the world's childhood deaths occur in this region, which also has unacceptably high rates of maternal deaths [1,2]. In addition, South Asia is also facing a growing burden of cardiovascular disease (CVD)[3]. South Asians, even if they live in developed countries, have substantially higher morbidity and mortality rates than the local majority populations from CVD. Further, compared to native Caucasian populations, migrants of South Asian descent have substantially poorer self-rated health (SRH)[4] which is a robust predictor of key health outcomes, including mortality and morbidity [5]. Out of all South Asian ethnic subgroups in the UK, Pakistanis and Bangladeshis have the poorest SRH[4]. This is particularly striking, as South Asians largely migrate to the UK in search of employment, and should thus be subject to the healthy migrant effect.

Despite its popularity in developed countries as a useful public health tool and its validation among migrant South Asian populations[4], the SRH scale has not been used at a population level in countries in South Asia. SRH was assessed for the first time at a population level in the National Health Survey of Pakistan (NHSP)-1990–94. We analysed NHSP data to estimate the prevalence of and identify risk factors for poor/fair SRH among individuals aged ≥15 years in Pakistan (n = 9442).

## Methods

NHSP's design is described in detail elsewhere[6,7]. Briefly, the NHSP was commissioned by the Pakistan Medical Research Council (PMRC), and conducted between 1990–1994, under the technical assistance of the United States National Center for Health Statistics. The design of the survey was a modification of United States' Third National Health and Nutrition Examination Survey (NHANES III). Using a two-stage stratified design, the NHSP was a nationwide household survey that collected information on health and nutritional indicators from a representative sample (18,135 individuals aged 6 months or older). Data collection involved the use of a questionnaire which had been validated in local languages. NHSP included an interview conducted at the participant's home by trained health workers, followed by a standardized physical examination in a well-equipped clinic by physicians.

Our primary outcome measure was SRH which was assessed using the question: "Would you say your health in general is excellent, very good, good, fair, or poor?" SRH was dichotomized into poor/fair and good (excellent, very good, or good). Household socioeconomic status (SES; high, middle or low) was defined on the basis of the number of material items owned (TV, bicycle, motorcycle, refrigerator, car, etc). Tobacco use (cigarettes, beddies, huqqa, chillum, and tobacco chewing) was dichotomized into current use or not. Respondents were asked, "Have you smoked at least 100 cigarettes or beddies during your entire life?" Those who replied "yes" were asked, "Do you smoke now?" Current smokers were persons who reported current smoking and having smoked at least 100 cigarettes or beddies during their lifetime. Respondents were also asked, "Have you chewed tobacco or used snuff at least 100 times during your entire life?" Those who said "yes" were asked, "Do you chew tobacco or use snuff now?" Those respondents who replied they did were reported as current tobacco chewers/snuff users.

Literacy was defined as the ability to read. There are four provinces in Pakistan: Punjab, Sindh, North West Frontier Province (NWFP) and Balochistan, each having its distinct culture, languages, and climate. Urban was defined as any area which had any of the following local government institutions at the time of 1981 population census: metropolitan corporation, municipal corporation, municipal committee, town committee, or cantonment board. All other areas were defined as rural.

**Figure 1:**
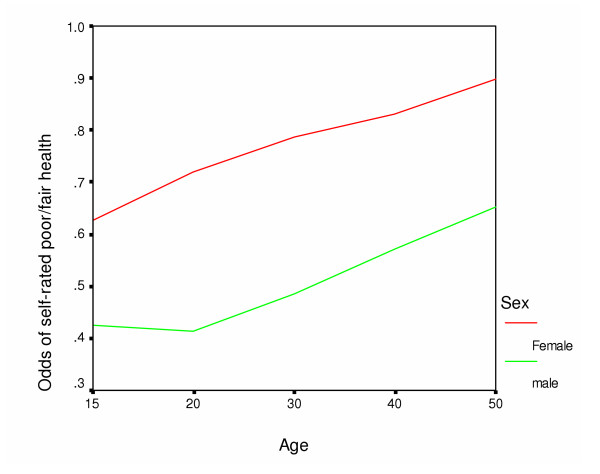
Graphic presentation of the association between poor/fair self-rated health and age by gender.

Analyses were performed with the SPSS statistical package (version 10.0). Means and standard deviations were computed for continuous variables, while proportions and 95% CIs for categorical variables. The association between poor/fair SRH and age, sex, literacy status, province of residence, area of residence (urban vs. rural), SES, marital status, hypertension, and current tobacco use was investigated, using univariate logistic regression. Variables that were associated with poor/fair health at p-value < 0.2 at the univariate logistic regression analysis were considered for the final multivariate logistic model (Table 2). Factors that were included in the final model were sex-age interaction, province of living, area of living (urban vs. rural), literacy, SES, and current tobacco use. Tests for interactions between sex and other independent variables were performed. The interaction between sex and age was assessed first using age as a continuous variable. However, based on the plot of association between poor/fair SRH and age by gender (Fig 1), age was used as a categorical variable (15–19 years and ≥20 years) in the final model.

**Table 2:** Unadjusted odds ratio for poor/fair self-rated health status for individuals aged 15 years and older in the first National Health Survey of Pakistan

**Variable**		**Total**	**Individuals with poor/fair SRH (%)**	**Unadjusted Odds ratio for poor/fair SRH (95% CI)**	**P-value**
**Number of individuals**		9373	6101 (65.1)		
					
**Age**	15–19	1636	866(14.2)	1.0	<0.0001
	≥ 20	7737	5235(85.8)	1.86(1.67,2.07)	
					
**Sex**	Female	4995	3856 (63.2)	3.22 (2.94, 3.51)	<0.0001
	Male	4378	2245 (36.8)	1.0	
					
**Marital status**	Never married	2239	1197 (20.6)	1.0	<0.0001
	Married	5980	4043 (69.4)	1.82 (1.65, 2.01)	
	Widow/widower/ divorced/separated	708	582 (10.0)	4.02 (3.26, 4.96)	
					
**Dwelling**	Rural	5995	4087 (67.0)	1.45 (1.33, 1.58)	<0.0001
	Urban	3378	2014 (33.0)	1.0	
					
**Literacy**	Literate	3012	1570 (25.7)	1.0	<0.0001
	Illiterate	6361	4531 (74.3)	2.27 (2.08, 2.49)	
					
**Socioeconomic status (SES)**	Low SES	3103	2228 (36.5)	1.81 (1.59, 2.04)	<0.0001
	Middle SES	4592	2891 (47.4)	1.21 (1.08, 1.35)	
	High SES	1678	982 (16.1)	1.0	
					
**Province**	Punjab	4858	4015 (65.8)	9.20 (8.17, 0.36)	<0.0001
	NWFP	1468	684 (11.2)	1.69 (1.47, 1.94)	
	Balochistan	1074	729 (11.9)	4.08 (3.48, 4.78)	
	Sindh	1973	673 (11.0)	1.0	
					
**Currently use tobacco**	Yes	2307	1433 (23.5)	0.84 (0.76, 0.93)	<0.01
	No	7066	4668 (76.5)	1.0	
					
**Hypertension**	Normal	7522	4832 (80.1)	1.0	<0.01
	Hypertensive	1772	1203 (19.9)	1.18 (1.05, 1.31)	

## Results

The overall agreement to be interviewed was 92.6%. A total of 9442 individuals interviewed were aged ≥15 years. The mean age (± SD) of the respondents was 36.3 years (± 17.1) [men: 37.3(± 17.9) vs. women 35.5(± 16.3)]. The mean missing data rate was 0.7 %. The missing data rates were 0 % for age, dwelling, literacy, SES, tobacco use and province; and 4.8 %, 0.9%, and 0.7 % for marital status, hypertension and self-rated health, respectively. Majority of the respondents lived in rural areas (64.1 %), were illiterate (68.0) and belonged to either low (33.1) or middle (49.0) socioeconomic group. As much as 7.6 % respondents (10.4 women vs. 4.6 % men) were widows, widowers, separated or divorced. Data on SRH were missing for 69 individuals (0.7%) so the final sample size was 9373 people. Of these, 65.1% respondents, 51.3 % men vs. 77.2 % women, rated their health as poor/fair (Table 1 and 2). In the Punjab province, 82.6 % people (69.6 % men vs. 94.6 % women) perceived their health to be poor/fair. Women had, at all ages, poorer SRH than men (Figure 1). Multiple logistic regression analysis showed a significant interaction between sex and age (p < 0.0001); but as shown in Fig 1 the interaction was due to the gender differences only in the ages 15–19 years, whereas poor/fair SRH at all older ages was more prevalent among women and increased at the same rate as it did among men. There was no significant interaction for ages 20 and above (p = 0.26). Women aged 15–19 years (adjusted OR 2.79, 95 % CI: 2.21–3.51), women aged ≥20 years (7.60, 6.23–9.27) and men aged ≥20 years(1.37, 1.14–1.64) were more likely to report having poor/fair health compared with men aged 15–19 years. Individuals living in Punjab had the highest odds (13.34, 95 % CI: 11.63–15.29) of poor/fair health. Other independent determinants of poor/fair health were low or middle SES, rural dwelling, literacy, and current tobacco use (Table 3).

**Table 1:** The distribution of self-related health status and other characteristics among men and women aged 15 years and older in the first National Health Survey of Pakistan

**Variable**		**Men (n = 4414) %**	**Women (n = 5028) %**	**All (n = 9442) %**
**Self-rated health status**	Excellent	0.6	0.3	0.4
	Very good	8.3	2.1	5.0
	Good	39.8	20.4	29.5
	Fair	37.0	39.4	38.3
	Poor	14.3	37.8	26.8
				
**Age**	<20	18.1	16.9	17.4
	20–29	23.8	27.1	25.5
	30–39	18.8	20.3	19.6
	40–49	13.3	14.4	13.9
	≥ 50	26.1	21.4	23.6
				
**Dwelling**	Rural	63.6	64.5	64.1
	Urban	36.4	35.5	35.9
				
**Literacy**	Illiterate	53.1	81.1	68.0
	Literate	46.9	18.9	32.0
				
**Socio Economic Status (SES)**	Low SES	33.7	32.6	33.1
	Middle SES	49.0	49.1	49.0
	High SES	17.3	18.3	17.9
				
**Currently use tobacco**	No	60.3	88.8	75.5
	Yes	39.7	11.2	24.5
				
**Province**	Sindh	20.7	21.2	20.9
	Punjab	53.3	50.9	52.0
	NWFP	15.5	15.7	15.6
	Balochistan	10.6	12.3	11.5
				
**Marital status**	Never married	31.2	19.6	25.1
	Married	64.1	69.5	66.9
	Widowed/widower	4.5	10.4	7.6
	Divorced	0.2	0.3	0.2
	Separated	0.1	0.2	0.2
				
**Hypertension**	Normal	79.8	82.0	80.9
	Hypertensive	20.2	18.0	19.1

**Table 3:** Adjusted odds ratio for poor/fair self-rated health status for individuals aged 15 years and older in the first National Health Survey of Pakistan

**Variable**		**Adjusted Odds ratio for poor/fair SRH (95% CI)**
**Male**	15–19 years	1.0
	≥ 20 years	1.37(1.14–1.64)
		
**Female**	15–19 years	2.79(2.21–3.51)
	≥ 20 years	7.60(6.23–9.27)
		
**Dwelling**	Rural	1.25(1.11–1.39)
	Urban	1.0
		
**Literacy**	Literate	1.33(1.17–1.50)
	Illiterate	1.0
		
**Socioeconomic status (SES)**	Low SES	1.56(1.34–1.82)
	Middle SES	1.17(1.02–1.35)
	High SES	1.0
		
**Province**	Punjab	13.34(11.63–15.29)
	NWFP	1.93(1.66–2.25)
	Balochistan	4.52(3.79–5.39)
	Sindh	1.0
		
**Currently use tobacco**	Yes	1.33(1.17–1.51)
	No	1.0

## Discussion

The prevalence of poor/fair health in Pakistan (65.1%) is far higher than that in Japan (9.8), Canada (11.7), United States (14.5) and the UK (25.3) in almost comparably aged populations [8-11]. However, it is closer to that obtained in a study in Russia in 1996 where the prevalence was higher, mainly because of major social changes and political and economic uncertainties after the collapse of the erstwhile Soviet Union [12].

South Asians, regardless of whether they live in their home countries or overseas have high morbidity and mortality rates. The fourth National Survey of Ethnic Minorities (1993–1994) in the UK that assessed the association between SRH and ethnicity found that people of Pakistani and Bangladeshi origin living in the UK had the poorest SRH, followed by Indians.

Our most striking finding was the substantially poorer health status, at all ages, of women compared to men in Pakistan. To our knowledge, this is the first study on SRH reporting a significant interaction between age and sex (p < 0.0001) – although the interaction was due to the gender differences only in the ages 15–19 years and not in older ages. For individuals aged ≥20 years, poor/fair SRH was more prevalent among women and increased at the same rate as it did among men. A limitation of the NSHP was that it did not assess mental health status, which is an important determinant of SRH. Studies in both developed and developing countries have shown than women have a higher prevalence of depression and anxiety than men. These disparities are strongly age-related. So differences in the rates of poor/fair SRH between women and men in our analysis may be due to possible age-related sex differences in the prevalence of common mental disorders in Pakistan. According to the WHO, an important reason for why women have higher rates of depression and anxiety than men is the high rates of domestic and sexual violence to which women are increasingly subjected [13]. Violence against women is endemic in Pakistan [14]as are mental disorders such as depression and anxiety [15].

The relationship between SES and the health status is well-established [16-18] although the potential for reverse causation (people may be poor because they are ill) makes such interpretations difficult. In the NHSP, SES was determined by the number of household items owned. In our analysis, even in the adjusted model people with low and middle SES were 1.56 and 1.17 times more likely to report poor/fair health compared with their counterparts with high SES. This is consistent with the findings of a study conducted in Poland and Hungary where as the number of household items owned increased, the odds of poor health decreased [19]. It is argued that material aspects of ownership of household items are important, but equally so may be the psychosocial aspects. For example, many household items not only have a direct protective effect on health, they also are marker of a higher status in the society. They may affect psychosocial well-being by psychosocial processes [20].

Another important finding is that tobacco use was independently associated with poor/fair SRH, although the cross-sectional design of the study limits the causal interpretation of this association. Several other studies have also reported a significant association between tobacco use and suboptimal SRH [21-23]. We also found province of dwelling and rural dwelling to be independently associated with poor/fair SRH.

## Conclusion

This is the first study reporting on the prevalence of and risk factor for poor/fair SRH at a population-level in a South Asian country. Our findings may be generalisable to several other countries in South Asia given similarities in physical environment, culture, socioeconomic conditions and public investment in health. The prevalence of poor/fair SRH in Pakistan is one of the worst ever reported globally, warranting immediate attention. Further research is needed to explain why women in Pakistan have, at all ages, poorer SRH than men.

## Competing interests

The author(s) declare that they have no competing interests.

## Authors' contributions

KA conceived the report, performed the statistical analysis, and drafted the manuscript. TJ and NC contributed to review, and to the revision of the report. All authors read and approved the final manuscript.

## Pre-publication history

The pre-publication history for this paper can be accessed here:


